# Retrograde transarterial closure of small aneurysmal perimembranous ventricular septal defects using the amplatzer duct occluder II: a single-center mid-term analysis

**DOI:** 10.3389/fcvm.2025.1659417

**Published:** 2025-10-07

**Authors:** Sibao Wang, Gang Luo, Zhixian Ji, Silin Pan

**Affiliations:** Heart Center, Women and Children’s Hospital, Qingdao University, Qingdao, China

**Keywords:** ventricular septal defect, aneurysmal, catheterization, amplatzer duct occluder II, retrograde transarterial approach

## Abstract

**Background:**

Transcatheter closure of aneurysmal perimembranous ventricular septal defects (pmVSDs) presents distinct anatomical challenges. While the retrograde transarterial approach using the Amplatzer Duct Occluder II (ADO II) has been described, data focusing on mid-term outcomes in this specific patient population remain limited.

**Methods:**

We conducted a single-center, retrospective analysis of pediatric patients with hemodynamically significant small aneurysmal pmVSDs who underwent transcatheter closure via a retrograde approach with the ADO II device. Primary endpoints included procedural success, complications, and mid-term clinical and echocardiographic outcomes.

**Results:**

Twenty-seven patients (median age 3.6 [3.0–4.6] years; median weight 15.9 [14.1–17.5] kg) were included. Defects had a median left ventricular inlet diameter of 4.0 [3.2–4.5] mm and right ventricular outlet diameter of 2.7 [2.5–3.1] mm; 59.3% were saccular aneurysms. The procedure was successful in 100% of cases using ADO II devices ranging from 4–4 to 6-6 mm. Median fluoroscopy time was 8.6 [8.1–12.1] min. No major procedural complications occurred. The immediate complete closure rate was 85.2%, rising to 100% at a median follow-up of 49 [28–72] months, with resolution of all clinical symptoms and no late device-related adverse events.

**Conclusion:**

Retrograde transarterial closure of small aneurysmal pmVSDs with the ADO II device is a feasible, safe, and effective therapeutic strategy in appropriately selected pediatric patients.

## 1 Introduction

Transcatheter closure has emerged as a well-established alternative to surgery for selected perimembranous ventricular septal defects (pmVSDs). Different devices with corresponding appropriate design features for the specific anatomy come into use, for example, the Amplatzer Duct Occluder II (ADO II) and the KONAR-MF™ VSD occluder (MFO) (1). The preferred procedural technique of choice—predominantly the classic anterograde (transvenous) or the retrograde (transarterial) approach—is a clinical matter of debate (2, 3). The anterograde approach often requires establishment of an arteriovenous (AV) loop, potentially making the procedure more cumbersome and leading to iatrogenic injury to the tricuspid apparatus. The retrograde approach offers a more coaxial, shorter route for the defect from the left ventricle, potentially easing the device deployment as well as reducing right-sided structure manipulation. This technique is not without its own risks, however, including potential interference with the aortic valve, a concern highlighted in recent reports (4, 5).

The participation of a septal aneurysm also complicates the anatomical landscape of pmVSDs, which influences the natural history of the defect and the transcatheter closure technical features.While extensive data exist for various devices and approaches, a need remains for focused reports on mid-term outcomes using the ADO II device via a retrograde approach, specifically for the challenging subset of small but hemodynamically significant aneurysmal pmVSDs. Therefore, this study aims to report our single-center experience, evaluating the safety, efficacy, and mid-term outcomes of this strategy as an additional contribution to the existing literature.

## 2 Methods

### 2.1 Study design and population

This single-center, retrospective cohort study included 27 consecutive children with aneurysmal pmVSDs who underwent transcatheter closure between January 2019 and October 2024 using a retrograde transarterial approach with the ADO II device. The study was approved by the Institutional Review Board of Women and Children's Hospital of Qingdao University (QFELL-KY-2019-64), and written informed consent was obtained from the legal guardians of all participants.

### 2.2 Pre-procedural evaluation and patient selection

Patients were considered eligible for the procedure based on the following criteria: (1) diagnosis of an aneurysmal pmVSD; (2) age >1 year with weight >10 kg; (3) a hemodynamically significant left-to-right shunt, evidenced by left heart volume overload on echocardiography or clinical symptoms such as congestive heart failure or recurrent respiratory infections; and (4) anatomical suitability for transcatheter closure, defined by a distance from the superior margin of the VSD to the aortic valve annulus of ≥2 mm and the absence of more-than-trivial aortic regurgitation or aortic valve prolapse. Exclusion criteria included a left ventricular defect diameter >6.0 mm, severe pulmonary hypertension, pre-existing significant atrioventricular conduction block, or active infection.

A critical selection criterion was the presence of a subaortic rim of at least 2 mm. This threshold is a widely accepted safety standard intended to mitigate the risk of iatrogenic aortic valve injury.It ensures a sufficient tissue margin for the stable anchoring of the device's left ventricular disc within the dynamic cardiac environment, preventing mechanical impingement on the adjacent, mobile aortic valve leaflets and subsequent aortic regurgitation (3, 6).

### 2.3 Procedure

#### 2.3.1 Catheterization procedure

Under general anesthesia, standard femoral arterial and venous access were established. After systemic heparinization, right and left heart catheterization was performed to measure pressures and determine the pulmonary-to-systemic blood flow ratio (Qp/Qs). Left ventriculography in a long-axis oblique view was used to characterize VSD morphology, shunt dimensions, and proximity to the aortic valve.

The defect was crossed from the left ventricle using a modified pigtail catheter and a hydrophilic guidewire. An appropriately sized delivery sheath was advanced over the wire, and the ADO II device (Abbott, Plymouth, MN, USA) was deployed. The detailed procedural steps for retrograde closure have been described previously (2). Device selection was based on angiographic measurements, with the device waist diameter typically exceeding the narrowest aneurysm neck diameter by 1–2 mm. Before release, final device position, stability, residual shunting, and valvular function were confirmed by both contrast injection and transthoracic echocardiography.

#### 2.3.2 Postoperative management and follow-up

Post-procedural care included oral aspirin (3–5 mg/kg daily) for six months. Patients underwent daily electrocardiography until discharge, with Holter monitoring and transthoracic echocardiography performed on postoperative day three. Follow-up evaluations, including echocardiography and electrocardiography, were scheduled at 1, 3, 6, and 12 months post-procedure and annually thereafter to monitor for complications such as device migration, arrhythmias, new-onset valvular regurgitation, and residual shunting.

### 2.4 Statistical analyses

Categorical variables are expressed as frequency and percentage. Continuous variables were assessed for normality and, being non-normally distributed, are presented as median [interquartile range]. The Wilcoxon signed-rank test was used to compare echocardiographic parameters between baseline and final follow-up. All statistical analyses were performed using IBM SPSS Statistics for Windows, Version 21.0 (IBM Corp., Armonk, NY, USA).

## 3 Results

### 3.1 Patient and defect characteristics

The baseline demographic, clinical, and anatomical characteristics of the 27 patients are summarized in Table 1. The median age was 3.6 [3.0–4.6] years, and the median weight was 15.9 [14.1–17.5] kg. The primary indication for closure in all 27 patients was a hemodynamically significant shunt, confirmed by objective evidence of left ventricular volume overload [median LVEDD Z-score 2.0 (1.7–2.2) at baseline]. Of the defects, 16 (59.3%) were saccular and 11 (40.7%) were multi-fenestrated. No patients had pre-existing aortic valve prolapse or more-than-trivial aortic regurgitation.

**Table 1:** Baseline, procedural, and hemodynamic characteristics of the study cohort (*n* = 27).

Variables	Values
Demographics	
Gender, n (%)	
Male	17 (63.0%)
Female	10 (37.0%)
Age（years）, median[IQR]	3.6 [3.0–4.6]
Weight（kg）, median[IQR]	15.9 [14.1–17.5]
Primary Indications for Closure, *n* (%)	
Congestive Heart Failure Symptoms	14 (51.9%)
Recurrent Respiratory Infections	17 (63.0%)
Left Heart Enlargement on Echo	27 (100%)
Defect Characteristics	
Aneurysm Type, *n* (%)	
Saccular	16 (59.3%)
Multi-fenestrated	11 (40.7%)
Echocardiographic Diameters (mm), median[IQR]	
VSD Inlet (LV side)	4.0 [3.2–4.5]
VSD Outlet (RV side)	2.7 [2.5–3.1]
Angiographic Diameters (mm), median[IQR]	
VSD Inlet (LV side)	4.4 [3.4–5.2]
VSD Outlet (RV side)	2.7 [2.5–2.9]
Hemodynamic & Procedural Data	
Qp/Qs, median[IQR]	1.55 [1.48–1.61]
Fluoroscopy Time (min), median[IQR]	8.6 [8.1–12.1]
ADO II Occluder size, *n* (%)	
4-4	3 (11.1%)
4-6	7 (25.9%)
5-6	9 (33.3%)
6-6	8 (29.6%)

IQR, interquartile range; LV, left ventricle; RV, right ventricle; Qp/Qs, pulmonary-to-systemic blood flow ratio; ADO II, Amplatzer Duct Occluder II; VSD, ventricular septal defect.

### 3.2 Procedural data

Successful ADO II device deployment via the retrograde arterial approach was achieved in all 27 patients. The median fluoroscopy time was 8.6 [8.1–12.1] minutes. No major procedural complications, such as device migration, peripheral vascular injury, or significant arrhythmia, were observed.

### 3.3 Postoperative care and immediate outcomes

All patients remained in sinus rhythm post-procedure. Immediate echocardiography confirmed appropriate device positioning with no new-onset valvular regurgitation. The immediate complete closure rate was 85.2% (23/27), with four patients having trivial residual shunts. Holter monitoring on postoperative day 3 revealed no clinically significant arrhythmias. Patients were discharged on oral aspirin for 6 months.

### 3.4 Mid-term follow-up and outcomes

The median follow-up duration was 49 [28–72] months. The trivial residual shunts in four patients had resolved by the 3-month follow-up, achieving a 100% complete closure rate for the cohort. No mortality, device embolization, infective endocarditis, or new-onset high-degree AV block occurred. All patients with pre-existing clinical symptoms (congestive heart failure or recurrent respiratory infections) experienced complete resolution. Echocardiographic assessment confirmed significant favorable cardiac reverse remodeling, with the median LVEDD Z-score decreasing from 2.0 [1.7–2.2] at baseline to 0.8 [0.5–1.1] at final follow-up (*p* < 0.001).

## 4 Discussion

This study demonstrates that retrograde transarterial closure of small aneurysmal pmVSDs using the ADO II device is a safe and highly effective strategy in a carefully selected pediatric cohort. Our approach achieved 100% procedural success, complete resolution of clinical symptoms, and significant favorable cardiac reverse remodeling. While watchful waiting is appropriate for many asymptomatic patients, our findings validate a proactive approach for the symptomatic subpopulation, where definitive closure effectively resolved adverse clinical and hemodynamic sequelae.

Our procedural outcomes compare favorably with those from larger series using different devices. The recent multicenter MIOS-MFO study, which evaluated the KONAR-MF occluder in 333 patients, reported a technical success rate of 97.6% and a 6-month closure success rate of 97.1% (1). Although our cohort is smaller and more homogenous, our 100% success rates for both implantation and complete closure at follow-up underscore the high efficacy of the retrograde ADO II technique within our carefully selected niche. This suggests that with meticulous patient selection, outstanding results can be achieved, complementing the findings from broader, more heterogeneous “real-world” registries.

The retrograde approach circumvents the need to establish an AV loop, thereby simplifying the procedure and minimizing the risk of iatrogenic injury to the tricuspid valve chordae, a notable concern with the conventional antegrade technique (7, 8). While this approach effectively mitigates the risks of the AV loop, it is noteworthy that newer, purely transvenous antegrade techniques are also being developed to achieve similar goals, representing a paradigm shift in VSD closure strategies (9). In our series, the retrograde approach was successful in cases where the venous sheath could not be advanced due to a large or acute angle between the right ventricular opening and the base of the ventricular septum. The elimination of arteriovenous track establishment reduces procedural complexity and minimizes the risk of iatrogenic injury to cardiac valves. However, this technique is not without potential pitfalls. Reports have raised caution regarding severe aortic valve injury, where the device or delivery system can interfere with leaflet function, leading to significant aortic regurgitation (4). Furthermore, late device embolization, although rare, remains a serious concern, particularly in small infants or in cases with compromised anatomy where suboptimal device seating may occur (10). Our excellent safety profile, with no instances of these major complications, is likely attributable to our stringent patient selection criteria, particularly the exclusion of patients with inadequate subaortic rims or pre-existing aortic valve abnormalities.

A salient finding of our study is the absence of complete AV block during follow-up, a significant improvement over the 1%–5% rates reported in historical series. This excellent safety profile is likely enhanced by the protective nature of the septal aneurysm itself. By deploying the occluder's left disc securely within the aneurysmal sac, a stable position is achieved while maintaining a crucial distance from the AV conduction system. This mechanism, where the aneurysm shields the conduction tissue from direct mechanical compression, has been proposed as a key factor in reducing the risk of heart block. Similarly, no new-onset or worsening aortic regurgitation was observed, a notable outcome given that its incidence can range from 3.3% to 11.0% (11–13). This favorable result is strongly associated with our rigorous pre-procedural anatomical assessment and exclusion of patients with pre-existing risk factors.

**Figure 1:**
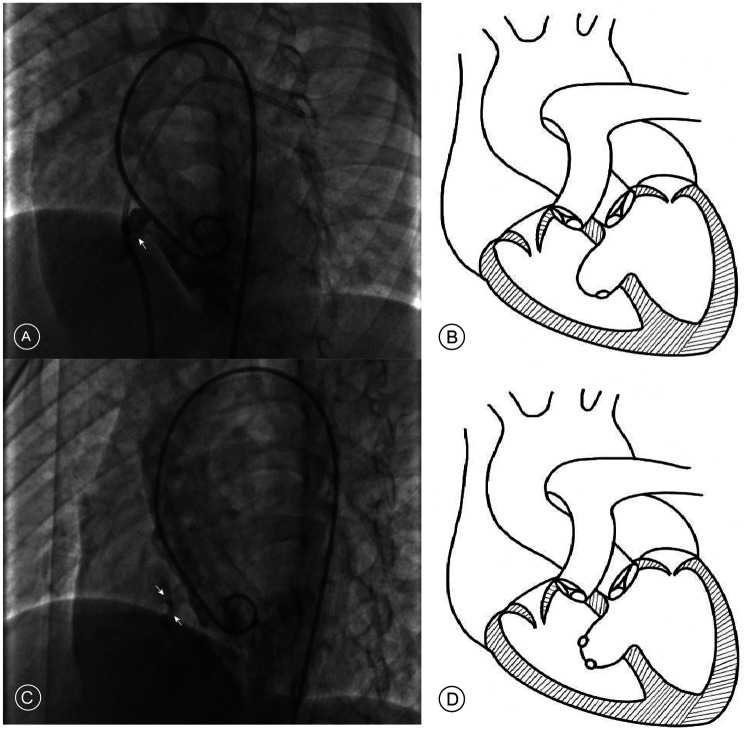
Angiographic findings and schematic diagrams of aneurysmal pmVSD. **(A)** Left ventriculogram in a long-axis oblique view showing a saccular aneurysm where the exit to the right ventricle forms an acute angle (white arrow). **(B)** Corresponding schematic diagram illustrating the acute angle. **(C)** Left ventriculogram showing a multi-fenestrated or “sieve-like” aneurysm, identified by multiple small contrast jets exiting into the right ventricle (white arrows). **(D)** Corresponding schematic diagram of a multi-fenestrated aneurysm.

The management of aneurysmal pmVSDs requires specialized consideration, as the aneurysmal morphology fundamentally alters the defect's three-dimensional geometry. This creates complex configurations that can render standard transvenous approaches challenging. For instance, in saccular aneurysms with an acute angle at the right ventricular opening, anterograde systems struggle to achieve proper alignment, a difficulty the retrograde approach accommodates more easily (Figures 1A,B). In cases of multi-fenestrated or “sieve-like” aneurysms, the retrograde route allows for superior device manipulation to ensure the left disc completely covers the septal base, preventing residual shunting (Figures 1C,D). Furthermore, the retrograde approach provides a more direct pathway through tortuous, tunnel-type defects and critically avoids the risk of entangling the tricuspid valve apparatus when adhesions are present.

### 4.1 Future perspectives in pmVSD management

The practice of transcatheter pmVSD closure moves towards the establishment of less harmful and more effective therapies (14). The development of new devices, for example, fully bioresorbable scaffolds, may eliminate the long-term risk of implant-related complications such as thrombosis or erosion. Concurrently, a debate also exists regarding the optimal time and even the need for intervention. Outcomes from the French nationwide cohort database FRANCISCO suggest the selection of closing hemodynamically significant pmVSDs often takes place according to particular anatomical characteristics or by the development of symptoms, rather than a protocol (15). This highlights that for a subset of patients, a conservative “watchful waiting” strategy remains a valid clinical option, underscoring the importance of individualized treatment decisions.

### 4.2 Limitations

The study is subject to several limitations. Its single-center, retrospective design inherently introduces the potential for selection bias. It is also crucial to acknowledge that our excellent outcomes were achieved in a highly selected patient population; consequently, the findings may not be directly generalizable to a broader, “all-comers” cohort. The stringent inclusion criteria, while vital for enhancing procedural safety, further limit the applicability of these findings. The study also lacks a direct comparative group treated with a conventional antegrade approach, precluding definitive conclusions about the superiority of the retrograde technique. Finally, all procedures were performed by experienced operators, and outcomes may not be fully reproducible in centers with less specialized expertise.

## 5 Conclusions

In conclusion, for pediatric patients with a hemodynamically significant small aneurysmal pmVSD, retrograde transarterial closure with the ADO II device is a safe and highly effective therapeutic strategy. When performed in specialized centers with careful patient selection, this approach yields excellent procedural success, durable resolution of clinical symptoms, and favorable cardiac reverse remodeling with a minimal complication profile.

## Data Availability

The original contributions presented in the study are included in the article/Supplementary Material, further inquiries can be directed to the corresponding author.
